# Immunology of AAV-Mediated Gene Transfer in the Eye

**DOI:** 10.3389/fimmu.2013.00261

**Published:** 2013-08-30

**Authors:** Keirnan Willett, Jean Bennett

**Affiliations:** ^1^Department of Ophthalmology, Scheie Eye Institute, F.M. Kirby Center for Molecular Ophthalmology, University of Pennsylvania, Philadelphia, PA, USA

**Keywords:** adeno-associated virus, RPE65, gene therapy for rare diseases, immune privilege, translational medical research, sub-retinal injection

## Abstract

The eye has been at the forefront of translational gene therapy largely owing to suitable disease targets, anatomic accessibility, and well-studied immunologic privilege. These advantages have fostered research culminating in several clinical trials and adeno-associated virus (AAV) has emerged as the vector of choice for many ocular therapies. Pre-clinical and clinical investigations have assessed the humoral and cellular immune responses to a variety of naturally occurring and engineered AAV serotypes as well as their delivered transgenes and these data have been correlated to potential clinical sequelae. Encouragingly, AAV appears safe and effective with clinical follow-up surpassing 5 years in some studies. As disease targets continue to expand for AAV in the eye, thorough and deliberate assessment of immunologic safety is critical. With careful study, the development of these technologies should concurrently inform the biology of the ocular immune response.

## Introduction

Visual impairment is a considerable burden to society. By the estimates of disability-adjusted life years, visual disorders and related diseases are comparable to diarrheal illness and HIV/AIDS when measured globally (1). While the majority of blindness in the world is avoidable by either prevention or therapy, little progress has been made for the remaining etiologies, many of which stem from well described genetic lesions. Gene transfer therapy has advanced tremendously in recent decades, and achieved a milestone success with the clinical efficacy of adeno-associated virus (AAV) mediated gene augmentation in the eye for Leber Congenital Amaurosis type 2 (2–4).

**Figure 1:**
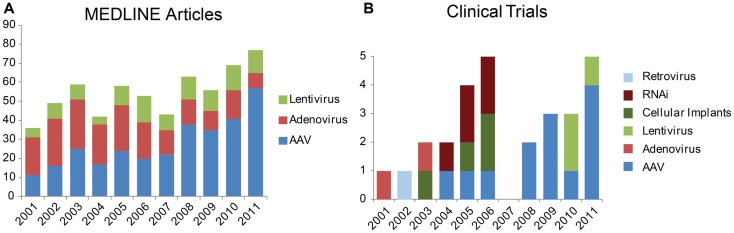
**Trends in vectors studied for gene therapy in the eye**. **(A)** Number of results returned when the MEDLINE database was queried via PubMed for “aav eye 2001” etc. **(B)** Newly registered clinical trials by year from the database Gene Therapy Clinical Trials Online from the Journal of Gene Medicine. Results restricted to “ocular diseases” and sorted by date approved.

**Figure 2:**
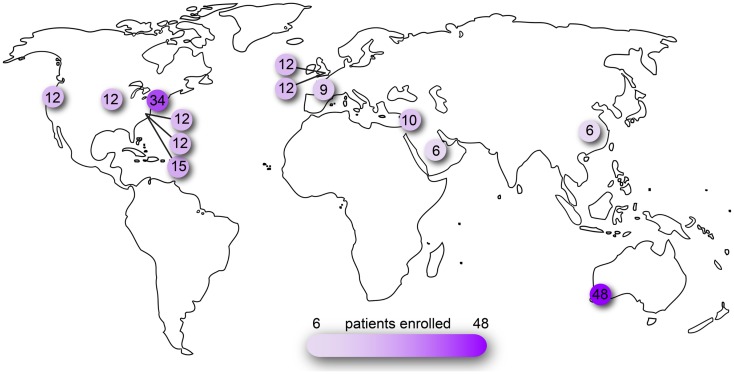
**Clinical trials of ocular AAV registered on www.clinicaltrials.gov**. Searched as search term = eye and interventions = adeno-associated virus OR AAV OR rAAV. Number of patients reported as actual or planned numbers for enrollment.

Other viruses such as lentivirus and adenovirus have been or are currently under investigation for ocular gene delivery. Compared to these viral and also non-viral modes of gene transfer, recombinant AAV continues to be a popular vector used in the eye both in basic science and translational studies (Figure 1). At present, clinical trials involving ocular administration of AAV are ongoing on four continents with an aggregate enrollment of over 200 participants (Figure 2). AAV, a helper-dependent single-stranded DNA parvovirus has never been shown to cause disease in humans or animals. It is appealing as a vector because it can stably and efficiently induce gene expression in dividing or terminally differentiated cells, has a favorable toxicity profile and benign immune response. Also, manipulation of the AAV capsid as well as promoters in the cDNA transgene effectively modulate cellular tropism which is critical to the cell-specific pathophysiology of many eye diseases (5).

**Figure 3:**
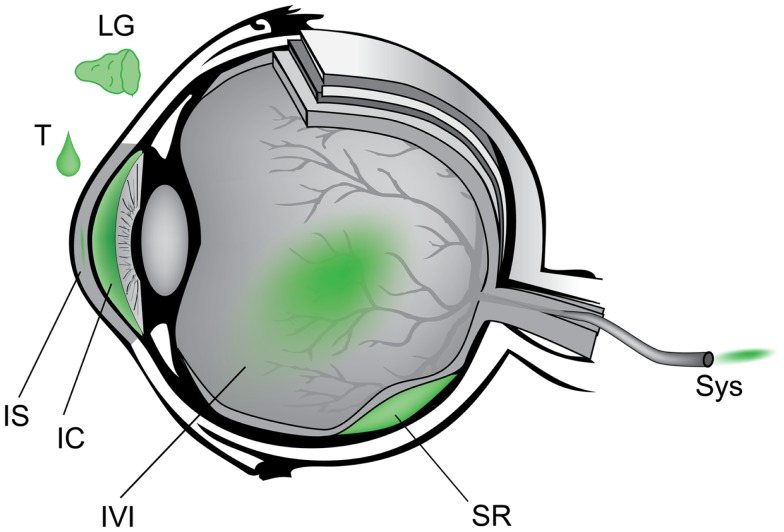
**Schematic of anatomic approach to administration of gene therapy vectors in the eye**. Counterclockwise from upper left – LG, lacrimal gland; T, topical eye drop; IS, intra-stromal of cornea; IC, intra-cameral, i.e., anterior chamber; IVI, intravitreal; SR, sub-retinal; Sys, systemic.

Anatomically, the eye is highly compartmentalized and many routes of AAV administration have been studied to target either anterior or posterior tissues (Figure 3). For example, lacrimal gland injection (6), topical eye drops (7, 8), intra-stromal corneal injection (9), and intra-cameral injection (10) provide access to the ocular surface, cornea, and anterior chamber which are implicated in dry eye disease, corneal dystrophies, and glaucoma. Intravitreal and sub-retinal injections access the neurosensory retina and the underlying retinal pigment epithelium (RPE). Systemic administration of gene therapy reagents is a theoretical alternative to intraocular surgery that avoids the potential complications of sub-retinal injection. Intravenous or intramuscular administration during the neonatal period in animals has been shown to diffusely transduce the retina (11). A major limitation of this approach, however, is the very large increase in vector dose compared to intraocular delivery, although certain technical improvements could be investigated – for example, adaptation of chemotherapy delivery methods via super selective cannulation of the ophthalmic artery (12). The systemic strategy is further limited by the mature blood brain barrier, the many avascular regions of the eye, and the potential for a detrimental inflammation in the setting of a necessarily large antigen load. By contrast, each of the intraocular compartments requires a relatively small volume of injection and thus highly purified vector is effective in small doses (13). The transparency of the eye is also advantageous in that it affords non-invasive direct visualization of neural and vascular tissue as well as other critical eye structures – facilitating research in animal models and close follow-up in the clinic. Furthermore, the symmetry of disease progression in most hereditary retinal diseases allows one eye to be used as the experimental target and the other as a control in research studies.

Thus far, diseases of the retina have garnered the most interest among ocular targets for gene therapy and will be the focus of this review.

## Immunity in the Eye

To maintain the transparent structures required for vision, the eye has conserved a number of adaptations that selectively diminish a maximal immune response. For example, complement-fixing antibodies, neutrophils, and macrophages are generally excluded from the eye as potential collateral damage could lead to lesions and opacities. Anatomically, the retina is an extension of the central nervous system and is protected by a selective blood-retinal barrier (BRB) established by non-fenestrated capillaries in the retinal vasculature and tight junctions in the RPE. Also, the avascular nature of much of the eye and the lack of lymphatics draining the anterior chamber, vitreous cavity, or sub-retinal space further limit classical antigen presentation and immune response (14). If soluble or cell-associated molecules bypass these obstacles, antigens are subject to a so called “deviant” immune response in the eye. The anterior chamber is the most studied entry point of these responses, and was originally described by Nobel Laureate Peter Medawar in his study of corneal transplants and their survival without immunosuppression (15). Termed anterior-chamber associated immune deviation (ACAID), this phenomenon is classically characterized by the elimination of the delayed-type hypersensitivity (DTH) response to the introduced antigen. This response is mediated by a population of antigen-specific regulatory T cells (Treg) that are elaborated in a multi-stage process involving the eye as well as the spleen, and these mechanisms are thoroughly reviewed by Streilein (14). This population of Treg cells can be transferred to naïve animals which adopt suppression of the DTH. Not surprisingly, this mechanism of immunosuppression is of considerable interest for treatment of autoimmune diseases and reproducing the necessary cytokine environment *in vivo* or *in vitro* has been shown to effectively cultivate similar Treg populations that mitigate autoimmune encephalomyelitis in animal models (16). Similar to the anterior chamber, antigens introduced to the vitreous and sub-retinal space exhibit an analogous immune deviant response (14, 17).

The cytokine environment in each of these ocular compartments is thought to be critical for a deviant response. For example, transforming growth factor β 2 (TGFβ2) was the first such cytokine described and is present in the vitreous, retina, and aqueous humor. The retina additionally contains vasoactive intestinal peptide (VIP), and somatostatin (SOM) while the aqueous humor contains these as well as α-melanocyte-stimulating hormone (α-MSH) and calcitonin gene-related peptide (CGRP). While all of these molecules are thought to contribute to immunosuppression, their various combinations in separated ocular compartments may underlie the subtle differences in immune deviation between them (18).

Following antigen exposure, the cellular immune response in the eye is similarly modulated by cytokines. The T cell helper Type 1 (Th1) response, for example, is generally injurious to the ocular tissues. Th1 cells secrete Interferon γ (Ifn-γ) which in turn activates phagocytes and stimulates production of IgG2a – a complement-fixing class of antibodies which incur collateral damage. By contrast, Th2 responses are thought to be better tolerated in the eye and can suppress macrophage activation. They are characterized by the anti-inflammatory cytokines IL-4, IL-10, and IL-13 – a milieu that favors IgG1, IgG3, and IgG2b which do not fix complement proteins. It is unclear in the literature if this sometimes termed “cross regulatory” population of Th2 cells in fact overlaps with the Treg population. Both are thought to be elaborated in the environment of TGFβ and IL-10 and both are known to suppress phagocyte activation. Treg cells are CD25^+^ and FoxP3^+^ and contain sub-populations of CD4^+^ “afferent” cells that suppress initiation of Th1 cells and CD8^+^ “efferent” cells that suppress Th1 action (18).

The regulation of these immune responses is critical to the safety of gene transfer in the eye. An unchecked inflammatory response could potentially damage neural tissue such as the photoreceptors which are post-mitotic at birth and do not regenerate throughout life. The efficacy of gene transfer events similarly depends on immune tolerance to a viral vector as well as the products of a transgene that may be novel to the host organism. Research into gene transfer therapies, therefore, has attempted to describe both the cellular and humoral immune responses to gene augmentation in the eye in animal models and ongoing human trials.

## Assays for Measuring Immune Response to AAV

Several techniques have been commonly adopted to characterize the ocular immune response to AAV technologies. Humoral responses are typically assayed by enzyme-linked immunosorbent assay (ELISA) which provides quantitation of specific antibody production and can also be refined to sub-type classes of antibodies characteristic of certain immune pathways (19). These antibodies can be measured in sera as well as vitreous and aqueous humor, and a comparison known as the Goldmann–Witmer (GW) coefficient has historically been used to localize antibody production either inside or outside the eye (19, 20). In addition, an *in vitro* functional test of AAV transduction can be conducted in the presence of test sera, commonly called a neutralizing antibody (NAb) assay. In this experiment, serum dilutions that inhibit transduction can be compared, and reflect the presence of neutralizing factors – presumably antibodies – that inhibit AAV transduction.

Cellular immune responses can also be precipitated by AAV or transgene products. An enzyme-linked immunosorbent spot (ELISPOT) is often used to quantitate populations of leukocytes that are activated in response to epitopes of interest. Commonly, Ifn-γ production is used to define activation and generally reflects a Th1 type response, although other markers can be used (21). Recent studies have also monitored cellular immune responses by flow cytometry detecting activation markers within the CD4^+^ and CD8^+^ compartments, such as Ki67, HLA-DR, and Bcl-2 (22, 23), however this method does not allow epitope characterization using specific peptide pools.

Finally, in animal studies, histological changes also reflect local immune responses in tissues of interest. Glial cell proliferation is one reaction to CNS insult and can be visualized by immunohistochemical probes detecting Glial fibrillary acidic protein (GFAP) (24, 25). Also, infiltrating leukocytes including activated macrophages can be visualized with antibodies against CD45, CD68, Iba1, and others (19, 24).

## Clinical Trials for Retinal Degeneration

Four independent groups have published results of safety and efficacy in clinical trials of gene augmentation with AAV2 for patients deficient in the isomerase RPE-specific 65 kDa protein (RPE65) (2–4, 26). The gene encoding RPE65 is one of at least 18 genes, when mutated, known to cause the rapid retinal degeneration known as Leber Congenital Amaurosis^1^. Some of the initial trials excluded patients with null mutations to remove the possibility of the introduced RPE65 protein being recognized as non-self (4), however some did not make this exclusion (2). Safety from an immunologic standpoint was assessed clinically as well as through laboratory evaluation.

Follow-up has surpassed 5 years for the first three groups and to date no major adverse events have been reported (27–29). By clinical exam, no significant inflammatory response has been attributed to the AAV or transgene product. Biodistribution studies have been part of each of the three initial trials and utilized polymerase chain reaction of AAV sequences in various compartments including tears, serum, saliva, and semen. Results have generally been negative with the exception of transient positivity in serum and tears which resolved within a few post-operative days.

To assess adaptive humoral response to the AAV2 capsid, functional assays of NAbs were performed. Generally, NAb assays were negative with the exception of one patient in the study by Maguire et al. (2) who experienced an increase in NAb titer which then decreased to a level slightly above baseline. In the Hauswirth et al. study (3), NAb titers were not measured functionally, but antibodies as assessed by ELISA were positive in one subject transiently. In the eye these responses have been much less than those induced by systemically injected AAV by several log units (30). Adaptive humoral responses to the transgene were assessed by serum ELISA in the Maguire and Bainbridge studies and results were negative (2, 4, 31).

Adaptive cellular immunity to the AAV2 capsid measured via ELISPOT was negative in each of the three seminal studies (2–4). Additionally, Hauswirth et al. assayed antigen-specific lymphocyte proliferation response (ASR) by thymidine uptake following exposure to AAV2 antigen, which was negative initially and at 1 year of follow-up (3, 32). Finally, adaptive cellular immunity against the delivered RPE65 gene product was assayed by Bennett et al. via ELISPOT and were generally negative with two exceptions which are presumed to be artifact (2, 31). The benign immunologic results of sub-retinal re-administration of AAV2.hRPE65 are discussed later in this report (19).

## Expanding Retinal Degeneration Targets

Many inherited retinal degenerations (RD) that were first characterized clinically now have a described genetic etiology and relevant testing is becoming cheaper and more available to clinicians. The national eye institute (NEI) now provides a program called eyeGENE that tests for known heritable eye diseases and compiles results into a database that is freely available to researchers and clinicians (33). Perhaps the most amenable of these genetic targets are autosomal recessive (AR) diseases that confer a loss-of-function mutation which can be potentially compensated by gene augmentation. As discussed above, the first of these specific mutations to be targeted for gene therapy in humans was the AR LCA2. This was an appealing initial target because (1) the 1.6 kB transgene is small enough to fit in the AAV2 capsid (<4.7 kB), (2) mouse and canine models of the disease are available, (3) the degenerative component in this particular disease is slow, thereby providing a wide therapeutic window, and (4) the primary cells effected are RPE which are efficiently transduced by sub-retinal injection of AAV2. LCA2 is a rare disease, however, with an incidence of ∼1:200,000, which amounts to an estimated 500 cases in the United States.

Proof-of-concept of gene augmentation therapy has been demonstrated in animals without significant inflammatory response in a number of other recessive somatic and X-linked RD targets using recombinant viruses including oculocutaneous albinism (34), x-linked juvenile retinoschisis (XLRS) (35, 36), and achromatopsia (37, 38). The retinitis pigmentosa (RP) phenotype encompasses>100 mutations, some of which include the LCA phenotype by certain nomenclatures. In this category, several AR gene targets have been similarly validated in animal models for possible gene augmentation: RPGR (39), GC1 (40), RPGRIP1 (41), MERTK (42), and AIPL1 (43). Furthermore, analogous studies have been done for Stargardt disease with mutations in ABCA4 using lentivirus (44) as well as Usher Syndrome Ib caused by mutations in MYO7A using AAV (45).

Also, toxic gain-of-function mutations characteristic of autosomal dominant disease can be targeted in a two-step approach – first by knocking down the defective gene with RNA interference (RNAi) then supplying a replacement molecule resistant to the introduced RNAi. This approach is being studied in mutations of rhodopsin (46, 47) and rds/peripherin (48). Alternatively, delivery of a wild-type molecule may be sufficient in some instances (49). So far there has not been any clear toxic effect of delivering rhodopsin or rds/peripherin in animals deficient in these proteins.

Delivery of generic pro-survival and anti-apoptotic factors has been investigated as a generalized treatment for a diverse set of retinal diseases, ranging from RP to achromatopsia, to macular degeneration^2^. AAV-mediated delivery of one such factor, ciliary-derived neurotrophic factor (CNTF) has been carried out in animal models with excellent success (50). In certain circumstances, however, it appears that the anatomical protective effect of CNTF can simultaneously diminish retinal function as measured by electrophysiology. Glial cell line-derived neurotrophic factor (GDNF) is a potential alternative that provides structural neuroprotection without adverse electrophysiologic effects at the same dose (51). Additional studies in cell and animal models have identified alternative neuroprotective agents, such as erythropoietin (EPO) (52), rod-derived ciliary neurotrophic factor (RdCVF) (53), and X-linked inhibitor of apoptosis (XIAP) (54). So far, there have not been significant toxic immune-related responses in animal models to these native proteins.

Retinoblastoma and other ocular neoplasms are also potential targets for gene therapy using AAV to deliver therapies that are not well tolerated systemically, such as the anti-cancer signaling protein interferon beta (Ifn-β) (55) or cytotoxic compounds (56). In mouse models, intravitreal injection of AAV2.Ifn-β showed anti-tumor effects and transgene expression was limited to the eye. No overt immune response was reported, and in these situations, some degree of immune activation could improve tumor regression.

## Anti-Angiogenesis

Retinopathies involving the vasculature are the leading cause of blindness in working-age and elderly adults in developed countries, comprising diabetic retinopathy (57) and the neovascular form of age-related macular degeneration (58), respectively. Efficacious treatment is now available in the form of molecules that inhibit vascular endothelial growth factor (VEGF), but their effects are temporary and require repeated intravitreal injections that can be inconvenient for patients and providers. The use of gene therapy to induce the production of anti-angiogenic molecules by endogenous cells could represent a durable solution for these common conditions.

Pigment epithelium derived factor (PEDF) is an attractive anti-angiogenic target because it opposes the action of VEGF and is also a pro-survival factor for retinal neurons. Extensive animal studies led to a clinical trial of adenovirus to deliver PEDF (59), and some mild to moderate inflammation was detected in 25% of patients but otherwise no adverse effects were noted. While adenovirus appears to have an acceptable safety profile in this study, the limited durability of the response favors AAV for future studies. Two additional trials currently underway employ AAV2 to deliver the soluble VEGF receptor sFlt intravitreally (in one study) and sub-retinally (in the other) (see text footnote 2). Non-human primate data supporting the intravitreal AAV2.sFlt trial (60) assessed immunologic responses and found mild to moderate effects evident on clinical exam as well as laboratory testing including induced antibodies to AAV2 in all animals. Time will tell if such effects are identified in humans.

## Methods to Enhance Efficiency

Several technical methods of enhancing the efficiency and specificity of AAV transduction in the eye have been investigated, such as use of cell-specific transgene promoters (61), engineered serotypes of AAV (24, 25), ultrasound micro bubbles (62), and co-administration of either chemo-therapeutic drugs (63) or adenovirus (64). It is unknown how these adjunctive techniques could affect the immune response in humans, but it could be speculated that additional antigens and disruption of cellular barriers could influence antigen presentation and immune infiltration. At present, gene transfer therapy in the human eye for LCA2 is not clearly limited by transduction efficiency, but as more indications are developed, the need for an adjuvant may become relevant. In situations where the dose of transgene is limiting, it could be preferable to increase the efficiency of viral transduction or modulate promoters to increase transgene protein expression, rather than increasing the dose of potentially immunogenic viral vectors. Similarly, it could be advantageous to use transgene constructs that are pharmacologically inducible, so that initial immune responses to surgical injury and viral capsids do not lead to bystander-induced immunity against therapeutic transgene products (65).

## Repeat Administration of AAV

A practical and ethical consideration for clinical trials of AAV in the eye is the timing of treatment of the contralateral eye. There is a theoretical concern that following exposure to AAV capsid or transgene during the initial treatment, the immune system could adopt memory. Thus, a subsequent injection in the contralateral eye could result in a primed immune system response that diminishes the efficacy of therapy, or worse, triggers destructive inflammation. An alternative theory supported by current data is that ocular gene therapy induces an immune deviant response analogous to ACAID as discussed earlier. In this model, the cytokine milieu of antigen presentation induces a systemic population of Treg which inhibit the cellular immune response to a second presentation of AAV or transgene. This ACAID-like response, however, varies with the ocular compartment injected. While sub-retinal injection seems to mirror the anterior chamber with respect to an immunosuppressive deviant response (17, 66), it has been shown that intravitreal injection of one eye can stimulate NAbs that diminish transduction events in the contralateral eye in animal models (67) – including novel AAV serotypes engineered to transduce the outer retina via an intravitreal injection (24).

Similarly, systemic re-administration of gene therapy vectors for other disease targets have demonstrated neutralization of transduction events due to preformed antibodies in several animal models including cystic fibrosis (68) and hemophilia B (69). In initial clinical trials for hemophilia B patients, therapeutic levels of the deficient coagulation factor IX were achieved, but only persisted ∼8 weeks (21). In this case, it seems that although patients with pre-existing neutralizing antibodies were excluded, a cellular response to AAV capsid incurred selective removal of transduced cells, and recent studies suggest that this limitation can be circumvented with certain immunosuppressive regimes (70). These immune responses to systemic administration in the presence of NAbs appear to differ from sub-retinal repeat administration possibly due to the immune-privileged and enclosed space of the sub-retinal compartment. Yet, other studies in non-ocular tissues have shown that despite the presence of NAbs, transduction events at systemic sites can still occur (71), underscoring the variability of response in different tissues and disease states.

In the eye, sub-retinal injection is the most thoroughly studied route for gene therapy and re-administration of AAV in this manner has been shown to be efficacious in affected dogs and safe in dogs and non-human primates (19, 72), as well as for three patients with 1 year of follow-up after re-administration (13). An alternative clinical strategy to avoid adaptation by the immune system would be to inject both eyes simultaneously. Bilateral surgery, however, incurs an increased risk to the patient’s residual vision in the event of a surgical complication. As a compromise, current bilateral studies in humans aim to operate on each eye 7–14 days apart, which is a short enough time to be considered one “event” by the immune system (see text footnote 2; NCT00999609).

## Conclusion

The eye has played a leading role in the clinical translation of gene transfer therapies. As the range of therapeutic targets increases in the eye, the immune response to these vectors and transgenes will continue to shape both efficacy and safety. The greater variety of tissues targeted as well as the sheer number of treated patients will likely reveal the diversity of immune responses possible in the eye which can further inform the way we study and execute these therapies.

While it is certainly advantageous that many parameters of these technologies can be engineered – surgical delivery, viral capsids, transgene cassettes etc. – it also complicates efforts to aggregate the safety data. For example, optogenetic therapy has been proposed for end-stage retinal disease (73). In this technique, simplified light-sensitive ion channels borrowed from *Archea* and plants could potentially be expressed in human retinal tissues. Clearly, the introduction of such foreign molecular patterns merits thorough study, even though the AAV vector has been shown to be generally safe.

Testing each technical modification for immune safety in animal models can be exhaustive and taxing on resources. However, given the capacity of the immune system for sensitive pattern detection as well as a potentially dangerous inflammatory response, it is critical that researchers remain vigilant in understanding the biology of the immune system and how it interfaces with these novel therapies.

## Conflict of Interest Statement

Keirnan Willett, none; Jean Bennett is a co-author on a patent, “Method of treating or retarding the development of blindness,” U.S. Patent 8,147,823 B2; April 3, 2012, but waived any potential financial gain. Jean Bennett is on the Scientific Advisory Board for Avalanche Technologies and is a founder of GenSight Biologics.

## Footnotes

^1^http://www.sph.uth.tmc.edu/RetNet

^2^www.clinicaltrials.gov
